# *RIL-Contour*: a Medical Imaging Dataset Annotation Tool for and with Deep Learning

**DOI:** 10.1007/s10278-019-00232-0

**Published:** 2019-05-14

**Authors:** Kenneth A. Philbrick, Alexander D. Weston, Zeynettin Akkus, Timothy L. Kline, Panagiotis Korfiatis, Tomas Sakinis, Petro Kostandy, Arunnit Boonrod, Atefeh Zeinoddini, Naoki Takahashi, Bradley J. Erickson

**Affiliations:** 1 Radiology Informatics Laboratory, Department of Radiology, Mayo Clinic, Rochester, MN USA; 2 Oslo University Hospital, Oslo, Norway; 3 Radiology Department, Khon Kaen University, Khon Kaen, 40002 Thailand

**Keywords:** Deep-learning, Medical image annotation, Annotation by iterative deep learing (AID), Segmentation, Classification, Software tools

## Abstract

Deep-learning algorithms typically fall within the domain of supervised artificial intelligence and are designed to “learn” from annotated data. Deep-learning models require large, diverse training datasets for optimal model convergence. The effort to curate these datasets is widely regarded as a barrier to the development of deep-learning systems. We developed *RIL-Contour* to accelerate medical image annotation for and with deep-learning. A major goal driving the development of the software was to create an environment which enables clinically oriented users to utilize deep-learning models to rapidly annotate medical imaging. *RIL-Contour* supports using fully automated deep-learning methods, semi-automated methods, and manual methods to annotate medical imaging with voxel and/or text annotations. To reduce annotation error, *RIL-Contour* promotes the standardization of image annotations across a dataset. *RIL-Contour* accelerates medical imaging annotation through the process of annotation by iterative deep learning (AID). The underlying concept of AID is to iteratively annotate, train, and utilize deep-learning models during the process of dataset annotation and model development. To enable this, *RIL-Contour* supports workflows in which multiple-image analysts annotate medical images, radiologists approve the annotations, and data scientists utilize these annotations to train deep-learning models. To automate the feedback loop between data scientists and image analysts, *RIL-Contour* provides mechanisms to enable data scientists to push deep newly trained deep-learning models to other users of the software. *RIL-Contour* and the AID methodology accelerate dataset annotation and model development by facilitating rapid collaboration between analysts, radiologists, and engineers.

## Introduction

Deep-learning algorithms typically fall within the domain of supervised artificial intelligence and are designed to “learn” from annotated data [1]. Deep-learning models require large, diverse training datasets for optimal model convergence. The ImageNet dataset used to train powerful general-purpose deep-learning image classifiers contains millions of unique images each annotated to describe the objects contained within the image [2]. While usually smaller, datasets used to train powerful medical image classifiers typically contain hundreds-to-thousands of annotated images [3–7]. The effort required to curate these training datasets is widely regarded as a major barrier to the development of deep-learning systems.

Numerous software tools have been developed to annotate medical imaging [8–18]. These tools commonly provide manual, semi-automated, and fully automated methods to label imaging. Semi-automated methods typically utilize traditional image processing techniques such as thresholding or edge detection [9, 10, 12, 19]. Fully automated methods are typically built upon semi-automated techniques and human-designed algorithms which encode domain-specific knowledge [13, 15, 17, 19]. Development of these algorithms is time consuming and the computational time associated with running many of them can be substantial.

Deep-learning algorithms “learn” to identify objects of interest in imaging data [1]. Utilizing deep-learning–based approaches for medical imaging annotation does not require the development of traditional human engineered algorithms. In many cases, deep-learning approaches to image analysis have been found to meet or exceed the performance of traditional algorithms [20, 21]. The computational time required to perform inference utilizing deep-learning models is often lower than traditional approaches. This suggests that implementing a deep-learning–based approach for dataset annotation may meet or exceed the performance of traditional human-designed annotation algorithms.

Medical image annotation software often does not provide tools that standardize the annotations used across datasets. Many annotation tools create annotations on an ad hoc basis. These tools place the burden of maintaining consistency in annotation labels on the analyst and have inspired efforts to standardize annotation lexicon [22]. Errors or variability in data annotation increases the size of the dataset required for deep-learning model convergence to a “correct” generalizable solution [23]. Errors specifically in the definition of the test dataset make it difficult to determine “true” model performance as model divergence from the test dataset may be appropriate.

Once created, annotation metadata must be associated in some fashion with the original imaging. Errors here result in annotation data loss and/or dataset corruption. The Digital Imaging and Communications in Medicine (DICOM) standard provides one solution to these challenges by enabling annotation metadata to be non-destructively embedded directly within medical imaging. This, however, alters the imaging files and can complicate using the same imaging for multiple annotation projects. Alternatively, if annotation data are not embedded within imaging then annotation metadata must be saved and associated in some fashion with the original imaging. Content management systems have historically provided a partial solution to these data management challenges. These systems provide database-like mechanisms to store and manage imaging and its associated metadata [24, 25]. However, annotation tools are usually stand-alone and not well integrated with content management systems. This lack of integration complicates workflows by requiring the image analyst to manage the movement of data between the content management system and the annotation software. The addition of these workflow steps results in the inability to guarantee that annotation metadata is correctly captured by a content management system.

## Software Overview

We developed *Radiology Informatics Laboratory Contour (RIL-Contour)* to accelerate medical image annotation for and with deep learning. A major goal driving the development of the software was to create an environment which enables clinically oriented users to focus on annotating imaging datasets using deep-learning methods and not on the underlying challenges associated with data transformation or management. Unlike annotation tools designed to annotate single images, *RIL-Contour* facilitates the consistent annotation of large medical imaging datasets required for developing deep-learning models and promotes collaborative dataset annotation by supporting concurrent multiuser workflows.

*RIL-Contour* defines voxel and imaging annotation definitions at the “dataset level” to enforce consistency of annotation definitions across all images in a dataset. This is similar to the concept of annotation template definitions used in other software [11]. *RIL-Contour* supports the use of deep-learning models to automatically perform voxel and text annotation of imaging. Additionally, *RIL-Contour* provides mechanisms to perform advanced deep-learning model visualization to aid image analysts and data scientists in understanding deep-learning models and provides methods to automate quantification of Dice and Jaccard metrics for deep-learning segmentation models.

*RIL-Contour* stores annotation metadata independently from imaging to enable imaging to be used in multiple annotation projects and to guarantee that the act of annotation does not alter image data files. *RIL-Contour* manages the storage of annotation metadata. While not common, other annotation tools provide similar functionality [11]. For datasets stored on a file system, *RIL-Contour* automatically maintains the file association between annotation metadata and imaging. Alternatively, *RIL-Contour* can be linked with a Medical Imaging Research Management and Associated Information Database (MIRMAID) content management system [24]. In this later configuration, *RIL-Contour* will silently retrieve imaging on demand and push and pull annotation metadata to and from the content management system.

### RIL-Contour User Interface

**Fig. 1:**
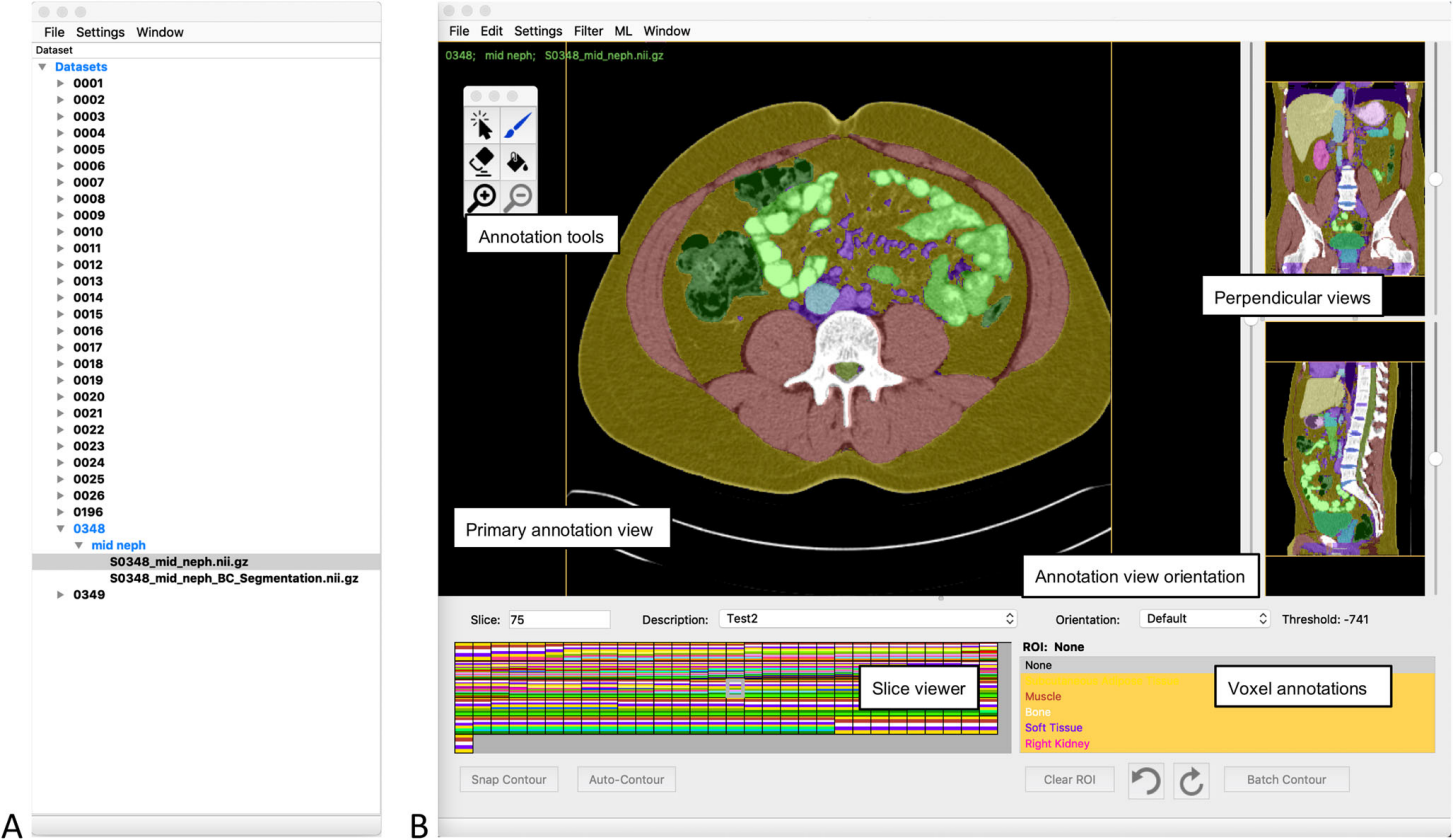
Screenshot of (**a**) dataset project viewer and (**b**) dataset annotation window. The dataset project viewer (**a**) shows a representative imaging project consisting of multiple data series, each consisting of one or more imaging exams, each further consisting of imaging series datasets. Imaging with annotation data is bolded and the selected series is shown in blue. The dataset annotation window (**b**) shows the currently selected dataset and the selected dataset’s annotations. Dataset slices in reference to the primary annotation view are shown in the slice viewer. Slice voxel annotations are indicated by the presence of one or more colors within the slice indicator. Voxel annotation along the axis perpendicular to the annotation view is shown to the right. The axis shown in the annotation view defaults to the projection with the greatest in slice voxel resolution and can be manually selected using the orientation view projection drop down.

Upon loading, *RIL-Conto*ur presents two windows, the dataset project viewer (Fig. 1(a)) and the dataset annotation window (Fig. 1(b)). The dataset project viewer displays a list of the imaging files associated with a project. The project viewer is designed to simplify the complexity of working with large datasets. From the user’s perspective, the dataset project viewer displays files in a hierarchy which mirror the datasets storage on the file system or for a content-managed dataset in a DICOM-inspired Patient → Study → Series hierarchy. The menus shown on the dataset project viewer window broadly provide access to dialogs which control project-wide settings (e.g., annotation definitions) and dialogs that perform operations across the project’s dataset (e.g., exporting data). Series which have been edited are bolded, allowing the user to quickly identify annotated imaging, and textual annotations associated with imaging can be shown as optional columns.

The dataset annotation window is the primary interface through which annotation is performed (Fig. 1(b)). *RIL-Contour* supports voxel annotations to define regions of interest (ROI) within images and text annotations to describe location-independent features or observations (e.g., image quality, presence of features, comments). *RIL-Contour* supports associating voxel ROI annotations with RadLex identification numbers (RadLex ID) to enable ROI definitions to be associated with a universally identifiable numerical nomenclature [22]. All dataset annotation operations are saved automatically as they are performed. For file system–based projects, the software supports versioning image annotations and supports common related versioning operations (e.g., viewing a version change history and rolling back to a previous version). To enable multiple users to utilize the same source imaging for independent annotation projects, *RIL-Contour* supports saving annotation data in an independent location on the file system or within a MIRMAID content management system [24].

### Voxel Annotations

**Fig. 2:**
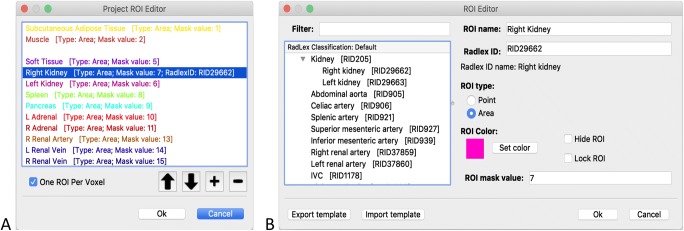
Screenshot of (**a**) ROI manager dialog window and (**b**) ROI editor dialog window. All existing ROIs defined for a project are shown in the project ROI editor window. ROI editor (**b**)—the editor window allows the user to change the name, RadLex ID, and color for any ROI.

**Fig. 3:**
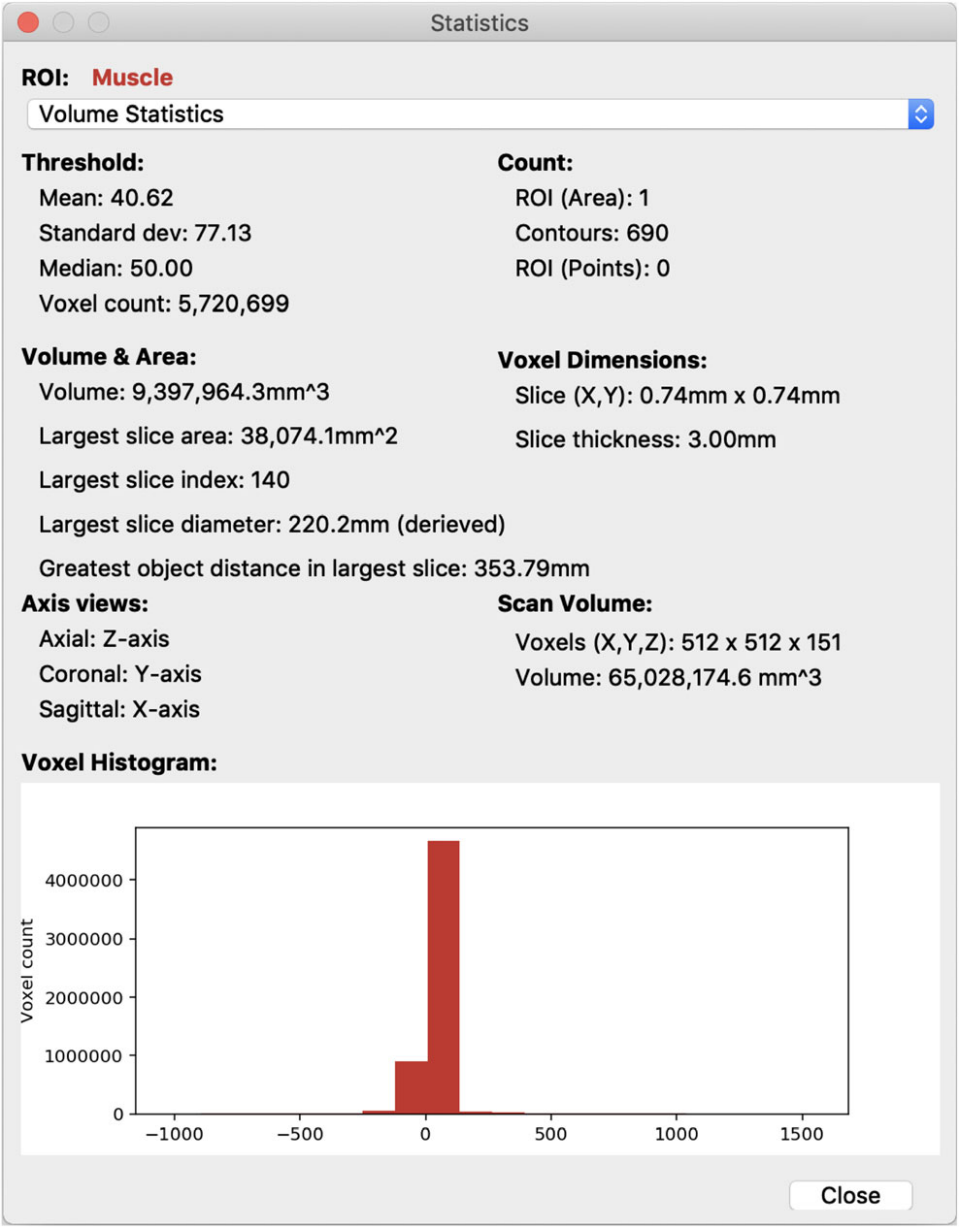
Statistics window displays descriptive statistics for the selected ROI with reference to the entire volume or selected slice.

*RIL-Contour* supports “area” and “point” voxel annotations to define ROIs within images (Fig. 2). Area annotations describe multi-voxel patches that can be used to either train an algorithm for segmentation or for classification. These annotations are often defined on multiple slices, and thus can represent multi-slice volumes. Point annotations describe the location of point(s) of interest within a series and can be used to define anatomical locations within a series or specifying the presence or absence of feature(s) within a slice. Descriptive statistics for a selected annotation can be shown through the statistics window (Fig. 3).

Manual definition of ROI is performed using the voxel annotation tools and filters. These tools and filters support common drawing operations (e.g., painting, erasing, filling, dilation, erosion, and undo/redo). *RIL-Contour* supports labeling voxels with multiple annotation labels, e.g., a voxel could be annotated as both kidney and tumor. Alternatively, the software can be set to enforce a one ROI per voxel mapping; e.g., a voxel could be annotated as a kidney or tumor but not both (Fig. 2). All manual segmentation tools support threshold-based application to selectively perform the desired annotation operation on voxels within a defined value range. The paint brush and eraser tools support cross-slice painting to automatically extend the operation to a predetermined number of adjacent slices. The histogram shown on the statistics window (Fig. 3) can be useful in determining the threshold range exhibited by a partially annotated tissue. The combination of threshold-based painting and multi-slice painting facilitates rapid manual segmentation of tissues which exhibit values which strongly differentiate them from surrounding structures. Finally, all ROI annotations support locking to prevent the ROI from being modified using manual, semi-automated, and fully automated deep-learning techniques.

**Fig. 4:**
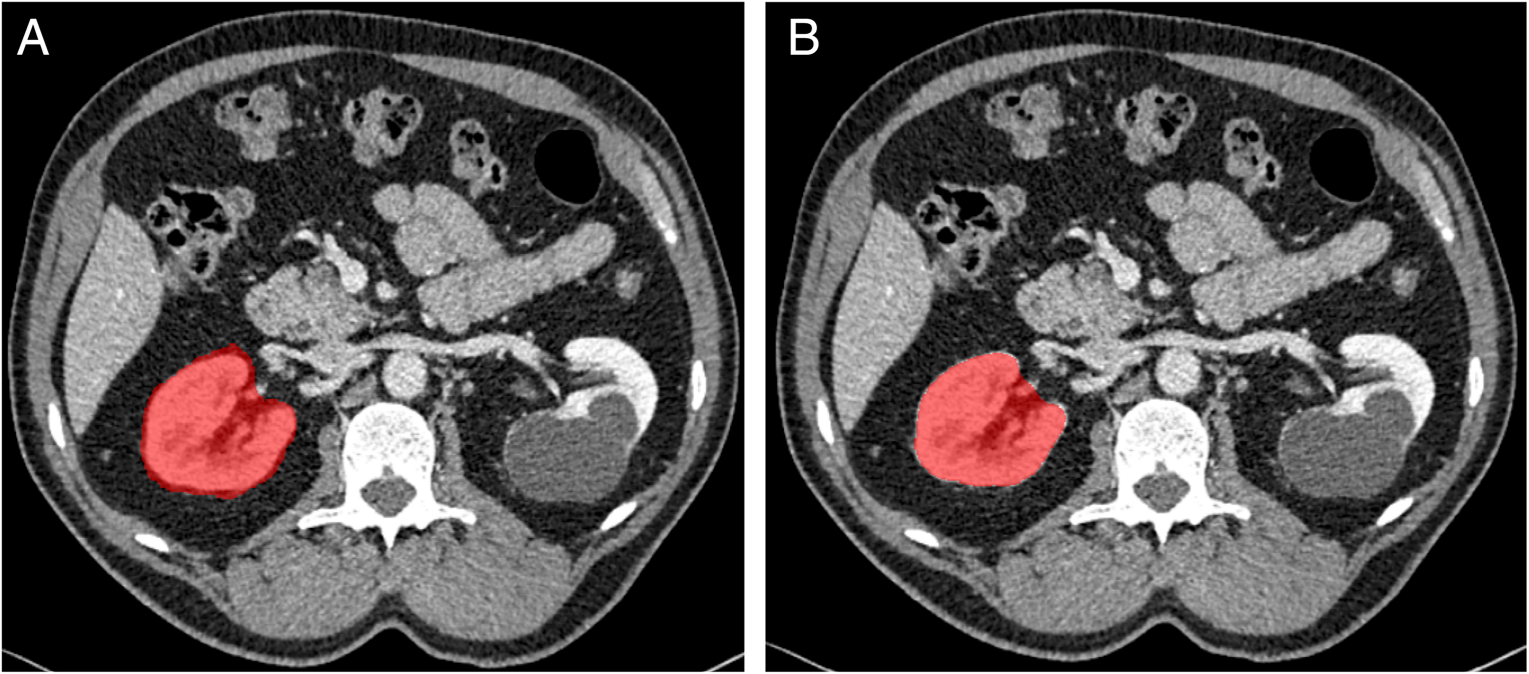
Example of semi-automated edge refinement of the kidney; (**a**) manual segmentation; (**b**) segmentation following semi-automated edge refinement.

*RIL-Contour* supports semi-automated ROI generation and edge refinement using the Minimal Interaction Rapid Organ Segmentation (MIROS) algorithm [26]. This algorithm was developed to refine the boundary of high-contrast organs (Fig. 4). Semi-automated edge refinement can be performed for a single slice using the “Snap Contour” and for multiple slices using the “Auto-Contouring” or “Batch Contouring” user interfaces (Fig. 1(b)). Slice segmentations generated wholly using semi-automated methods are illustrated within the slice viewer by a lighter version of the ROI’s color to differentiate them from user-edited annotations (Fig. 1(b)).

### Text Annotations

*RIL-Contour* supports descriptive text annotations to capture non-voxel-based observations. Text annotations can be restricted to a predefined set of values to standardize annotations. All text annotations can be shown as optional columns in the dataset project viewer to identify images in the dataset containing the text annotation.

### Import and Exporting Annotations

*RIL-Contour* supports importing and exporting ROI voxel annotation data to and from binary file masks. To define multiple overlapping ROI in a single binary voxel mask, files can be written out as the “binary or” of the overlapping ROI mask values. For masks exported to the file system, annotation masks are written in a hierarchy that mirrors the original dataset. Masks exported to the file system are accompanied by a data file describing the binary mask, e.g., mapping between the ROI mask value and a RadLex ID. Copies of the original input imaging and original *RIL-Contour* annotation data file can optionally also be written out. For content-managed workflows, binary masks can be exported back into a MIRMAID content management system or to the file system. Alternatively, descriptive statistics of voxel annotations and tables listing the text annotations associated with imaging can be exported in Excel (Microsoft, Redmond, WA) or comma-separated value (CSV) format.

### Concurrent User Annotation and Multiuser Workflows

**Fig. 5:**
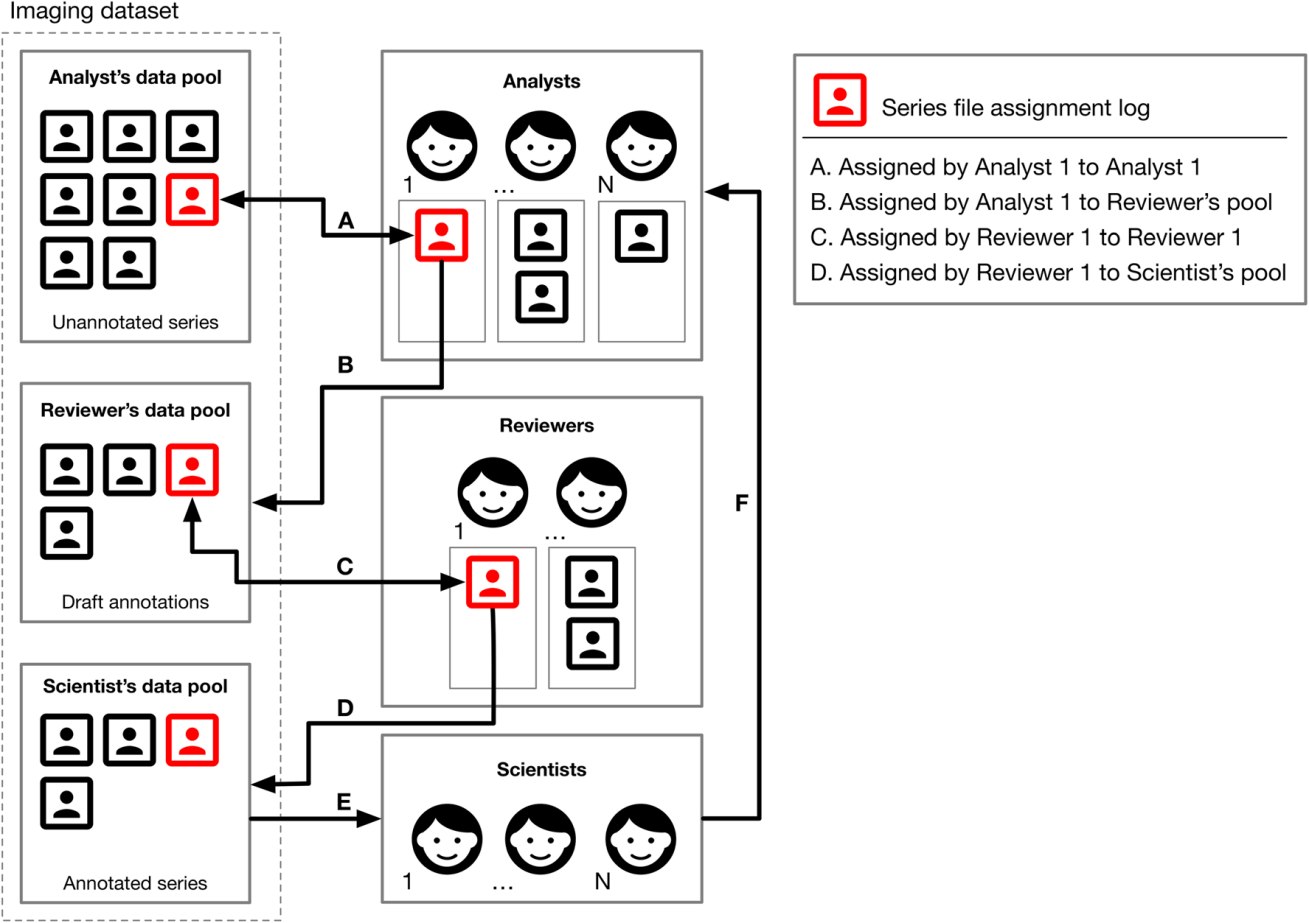
Example collaborative multiuser annotation workflow illustrating the controlled annotation of an individual series (red) by multiple users. Unannotated series assigned to analyst group at the start of the project. Analysts acquire unannotated series for annotation from the analyst pool. Analysts can (**a**) return partially annotated series to the Analyst’s pool for further editing by other analysts or (**b**) assign the annotated series to Reviewer’s pool; series can no longer be acquired by an analyst. (**c)** Reviewer 1 acquires the series from the Reviewer’s pool, if the annotations look correct, (**d**) reviewer assigns image to Scientist pool. Alternatively, not pictured, if the reviewer deemed the annotations poor, they could have re-assigned the series back to the analysts pool or to a specific analyst. (**e)** Scientists use available curated dataset to train deep-learning model. (**f)** Trained deep-learning model pushed to analysts to perform draft dataset annotation as an example of implementing the AID dataset annotation methodology.

*RIL-Contour* supports concurrent dataset annotation by multiple users. For datasets stored in a MIRMAID content management system, *RIL-Contour* utilizes locking mechanisms to enable multiple users to concurrently annotate independent imaging series. For datasets stored on the file system, *RIL-Contour* supports series locking and additionally supports multiuser workflows which define series-specific user-level rights to generate annotations for imaging and define the set of other software users to which a user can assign image annotation rights to. These workflows are designed to enable multiple people to work concurrently to annotate, review, and utilize the data for machine-learning purposes. Figure 5 illustrates an example annotation workflow in which multiple-image analysts generate annotations, the generated annotations are reviewed, and the resulting annotations are used by data scientists to train a deep-learning model. To support these workflows, *RIL-Contour* automatically versions series annotations when series ownership changes. *RIL-Contour* multiuser workflows are described in a YAML file which can be optionally embedded within a *RIL-Contour* project description file or specified at run time through a command line option.

### Deep-Learning Powered Annotation

*RIL-Contour* supports utilizing trained deep-learning models to perform fully automated annotation. *RIL-Contour* utilizes a “no-coding” plugin architecture to make it relatively simple to deploy deep-learning models in the software. The plugin interface is designed to run deep-learning models developed in Keras running on Tensorflow. The plugin execution framework instantiates models on demand. The time required to load a model is related to the model complexity. Once loaded, the computational costs associated with model inference for most models are typically low enough that models can be run on a standard modern CPU.

**Fig. 6:**
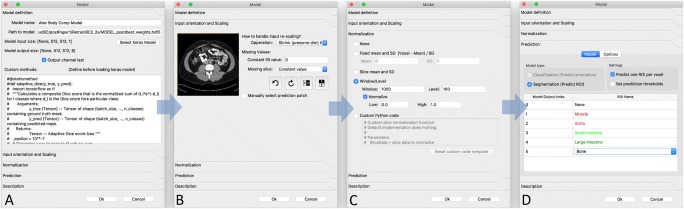
Importing a deep-learning model into *RIL-Contour*. Metadata required to load ML model is defined in the model wizard; (**a**) defines model name and loads model and weights (HDF5 file) and optionally defines custom python model loading code; (**b**) defines affine transformations required to transform slice input into the model; (**c**) defines image normalization to perform prior to model execution; and (**d**) links model output with custom *RIL-Contour* annotations.

*RIL-Contour* defines the preprocessing operations (e.g., normalization, mapping model output to annotation settings) required for model inference in metadata which it stores alongside an HDF5 file that describes the model’s weights and optionally architecture. To enable model metadata to be defined with little-to-no coding, *RIL-Contour* provides a model creation wizard that resides inside of a model manager dialog that steps users through the definition of the requisite metadata (Fig. 6).

The *RIL-Contour* model manager supports model versioning and model sharing. Model versioning is designed to enable models to be easily updated with a new set of learned weights and/or architecture while maintaining a history of prior model configurations. The software supports importing and exporting models with their definition metadata and has functions to automate model discovery to enable models to be automatically imported into the software as they are made available. This feature has been designed to enable data scientists to “push” new and updated deep-learning models to other users of the software (Fig. 5).

### Understanding Model Inference

**Fig. 7:**
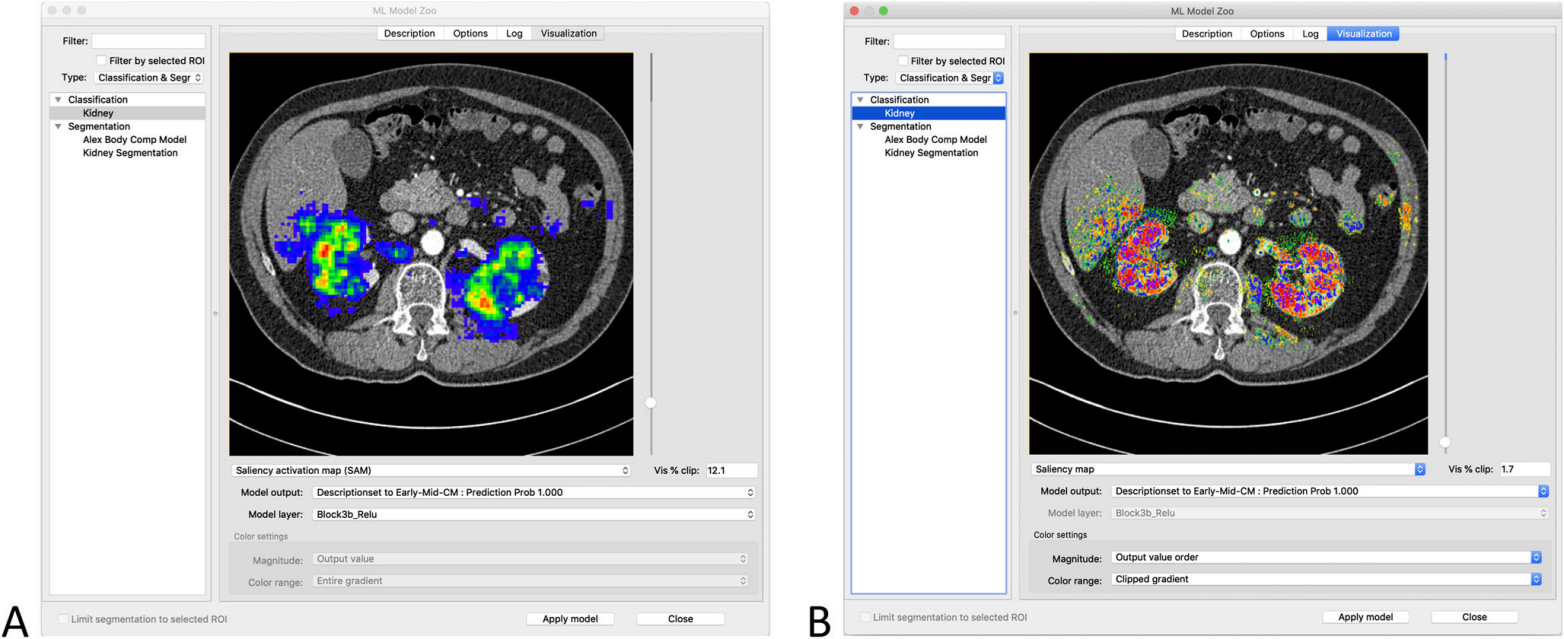
Representative interactive model visualizations generated in *RIL-Contour* illustrating the regions of an image that the model strongly activated on when performing inference; (**a**) saliency activation map (SAM); (**b**) saliency map visualizations of a deep-learning model designed to classify CT contrast enhancement. Visualizations shown using a rainbow color pallet; red = high, purple = low. This visualization indicates that portions of the left and right kidney are being used by the model to identify the imaging’s renal contrast enhancement phase.

*RIL-Contour* supports the interactive generation of visualizations which identify the regions of images that models identify when performing prediction (Fig. 7) [4]. The software supports a variety of state-of-the-art visualization approaches such as saliency maps, class activation maps (CAM), gradient class activation maps (Grad-CAM), and saliency activation maps (SAM) [4, 27–30]. These techniques allow analysts without a data science background to quickly and intuitively understand the regions of an image that a deep-learning model responds to. Each of the model visualization techniques employed within *RIL-Contour* generates an “activation” metrics for each voxel. To enable users to rapidly focus on meaningful regions of activation, *RIL-Contour* performs automatic thresholding to hide low-intensity background activations; this setting can also be dynamically adjusted by the analyst.

### Deep-Learning Model Segmentation Model Metrics

*RIL-Contour* supports automated quantification of Dice and Jacard segmentation metrics between a deep-learning model’s predictions and image segmentations defined in the software. Metrics are computed on a per-slice basis for slices selected in the software. Slice segmentations metrics are summarized as volume segmentation metrics.

### Annotation by Iterative Deep Learning

The time required to curate large datasets is a major roadblock to developing novel deep-learning models. *RIL-Contour* can accelerate data annotation through the process of annotation by iterative deep learning (AID). AID accelerates dataset annotation by utilizing deep-learning models to generate draft annotations. AID is based on the observation that it is typically faster for humans to edit or correct a good-but-not-perfect image annotation than to generate one entirely from scratch.

**Fig. 8:**
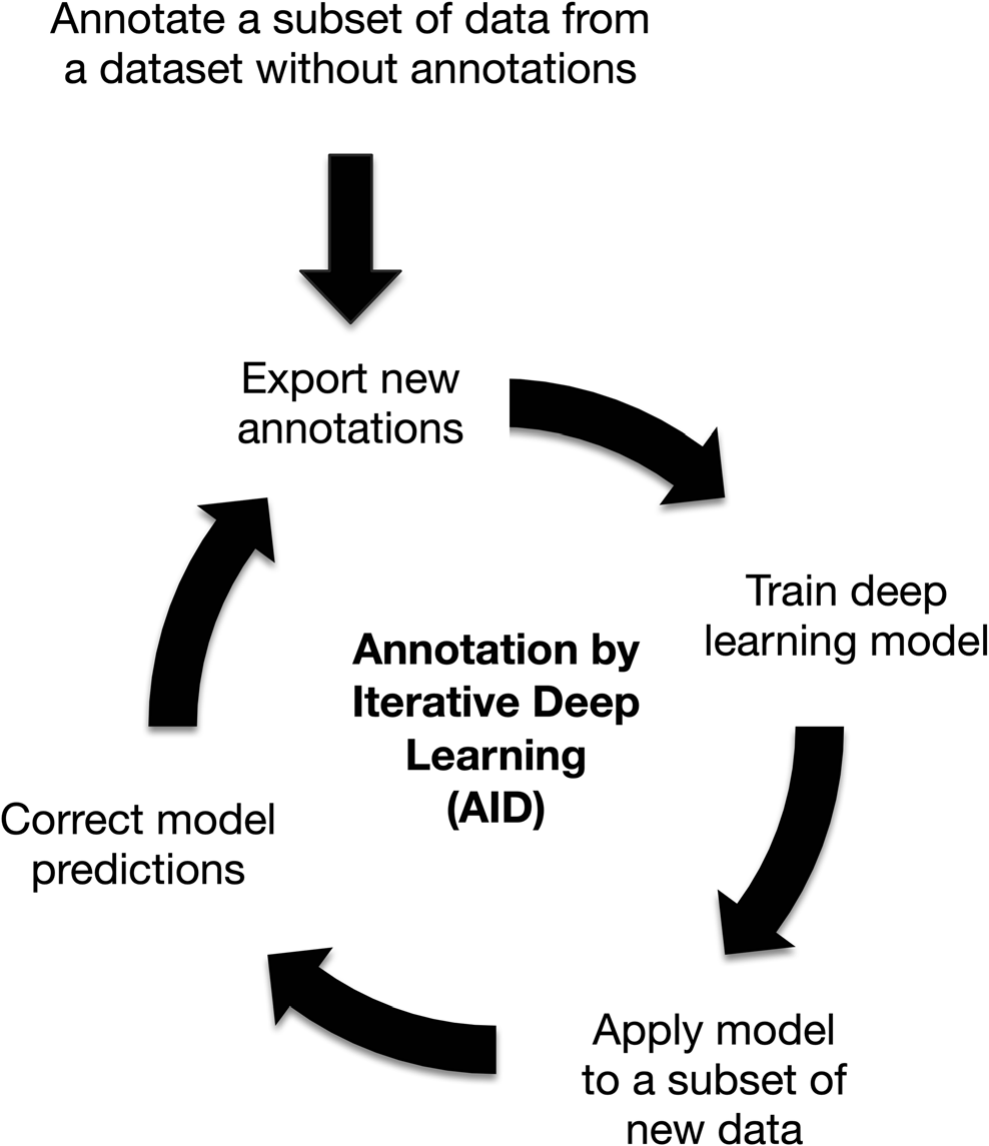
Annotation by iterative deep learning (AID).

Using the AID process, dataset annotation begins with an entirely unannotated dataset. From this, a small subset of the data is selected and annotated using traditional methods. This initial annotated dataset is then used to train a “development” deep-learning model to perform the desired annotation. This “development” model is then deployed from within *RIL-Contour* to generate draft annotations for the next set of training data. The newly created draft annotations are then corrected as necessary from within the *RIL-Contour* and the now expanded annotated dataset is exported from the software and used to train the next “development” model. This process is repeated iteratively until the entire dataset is annotated or until a model is created with sufficient accuracy that further iteration is no longer required. The AID methodology is illustrated in Figs. 5 and 8. Conceptually, AID is described as a cycle. However, given sufficient human resources, model development and dataset annotation can be conducted concurrently (Fig. 5); new models can be developed as new data becomes available and deep-learning annotation models can be utilized as they are created.

We have utilized *RIL-Contour* for multiple annotation projects. These projects have used the software for annotation of MRI, CT, and US imaging collected at the head, chest, and abdomen to generate annotations of brain, abdominal organs, tumors, and other tissues and to generate annotations that categorically classify the presence or absence of tumors in imaging or the contrast enhancement phase of a CT series. To date, we have used RIL-Contour to perform data annotation for over 12 projects. We report several case studies to illustrate how *RIL-Contour* can be used to accelerate medical image annotation.

Our largest project to date involves segmenting 35 unique organs and tissues in CT volumes of the abdomen. Project staff consists of 17 image analysts, 5 radiologists, and 3 data scientists who coordinate solely through *RIL-Contour*. Qualitatively, AID methodology greatly decreased the human time required to annotate new series for this project. Initially, starting from minimal base annotations, annotators required approximately 40 h to fully segment the abdominal organs in a series. At present, we have 99 annotated volumes annotated. The AID methodology has decreased average volume annotation time to approximal 8 h per series, 80% reduction in annotation time.

In another example, we created a novel dataset to train a deep-learning model to locate the vertebral bodies. Seven analysts utilized the software to define the desired anatomy. The entire project, which involved segmentation of 132 cases, took less than a week from conception to successful conclusion.

In another example, we utilized *RIL-Contour* to categorically annotate the contrast enhancement phase of abdominal CT imaging. Annotations were generated by 3 radiologists. Three thousand images were annotated. These annotations were used to train a contrast enhancement prediction model [4]. A *RIL-Contour* plugin for this model is shared on GitLab (see “Software Availability”).

We have found *RIL-Contour* to be a useful tool for deploying deep-learning models to collaborators who may have little-to-no experience with machine learning. In a recent example, we utilized *RIL-Contour* to correlate body composition, in particular visceral adiposity, with waist-hip measurements taken at our clinic. *RIL-Contour’s* no-coding interface allowed our collaborator, who had no experience coding, to utilize deep-learning models to perform automated segmentation after an hour of training.

## Discussion

The development of deep-learning models for medical imaging typically requires the annotation of hundreds-to-thousands of images [3–7]. This process is time consuming and potentially error prone. Software tools which facilitate rapid accurate image annotation and annotation review are needed to accelerate the development of deep-learning datasets and models.

*RIL-Contour* has been designed with the goals of accelerating the annotation of medical imaging for deep learning. *RIL-Contour* contour accomplishes this by (1) providing a tool that simplifies the challenges of working with large imagining datasets in a collaborative research environment, (2) by providing a tool that enables deep-learning models to be utilized directly from within the software to perform fully automated annotation, and (3) by providing a tool that facilitates the visualization of and understanding of deep-learning models.

Variability or errors in dataset annotation increase the size of the training dataset required for accurate deep-learning model convergence [23]. A strategy utilized by other medical imaging software has been to standardize definition of annotations across the images in a dataset using templates [11]. *RIL-Contour* adopts a similar strategy to ensure consistency in the definition of annotations in a dataset. This design paradigm guarantees that a given ROI will have the same name, RadLex ID, and voxel mask value for all images in a *RIL-Contour* dataset and that text annotations will fall within a predefined set of values.

Few medical imaging research annotation tools are designed to manage the association between imaging and annotation metadata when the metadata is not stored directly within the source imaging. A notable expectation is the work of Rubin et al. [11]. Content management systems such as *MIRMAID* and Extensible Neuroimaging Archive Toolkit (*XNAT)* provide systems to accomplish this [24, 25]. However, in working with most annotation software, these systems typically require the data analyst to manually move data between annotation software and the content management system. These additional steps add workflow complexity and are potentially error prone. *RIL-Contour* provides a mechanism to manage the association between imaging data and annotation metadata for datasets stored on the file system or within a MIRMAID content management system. These interfaces are designed to minimize workflow complexity and empower the data analyst to focus on data annotation and review and not on the management of imaging and metadata.

*RIL-Contour* is designed to simplify the application of deep-learning models for the purposes of medical image annotation. *RIL-Contour* utilizes a plugin engine to load and run deep-learning models at run time. The *RIL-Contour* engine supports models developed in *Keras* running on *Tensorflow*. Future support for additional platforms is planned. To execute a model, the plugin engine loads the model at run time, from source or an HDF5 file, normalizes and transforms the input imaging to match the model’s requirements, runs the model, and, for segmentations, transforms the model output into *RIL-Contour* voxel annotations. The plugin engine enables *RIL-Contour* to interact directly with models. This allows *RIL-Contour* to provide a graphical user interface (GUI) model definition wizard which walks users through the process of importing a deep-learning model based, in part, on the underlying architecture of the model and enables the software to provide model visualization features which rely on the ability to rewrite a model and compute the output and gradient of arbitrary model layers.

To our knowledge, *DeepInfer* is the only other medical image annotation tool developed to facilitate automated image annotation using deep learning [31]. *DeepInfer* is a *3D Slicer* plugin which enables *3D Slicer* to utilize deep-learning models to perform fully automated image annotation [9, 31]. In terms of functionality, *RIL-Contour* and *DeepInfer* both automate the application of deep-learning models for the purposes of data annotation. *DeepInfer* utilizes a Docker-based execution engine to run deep-learning models. Due to its Docker-based design, *DeepInfer* does not directly interact with models and as a result cannot directly perform the model modifications required for the generation of advanced visualizations.

The *RIL-Contour* plugin interface currently supports two-dimensional models and patch-wise application of three-dimensional models for segmentation or classification. Support for whole volume three-dimensional models is planned. The generation of CAM visualizations requires CAM-specific model architecture, within network SAM and Grad-CAM layer visualizations are supported for both convolutional and activation layers with non-linear activation functions [4, 27, 28, 30].

The effort required to curate training datasets for deep learning is widely regarded as a major barrier to the development of deep-learning models. Numerous groups have attempted to accelerate machine-learning model training through processes designed to optimize the creation of training datasets [32–34]. Deep-learning methods have been proposed to accelerate interactive segmentation and to propagate segmentations across slices [35]. Other techniques, auto-annotation and pseudo-annotation, utilize multiple instance learning to automatically identify meaningful annotations from a set of predetermined noisy labels; labels that both correctly and incorrectly label data [36–38].

Here, we propose the AID methodology to accelerate human-driven data annotation of medical imaging. AID is an example of how artificial intelligence can be used to augment and accelerate human performance while retaining human supervision. AID methodology is similar to a classification-based annotation system described for natural world images [32]. The underlying premise behind AID is that a machine-learning model can be used during the construction of a supervised training dataset and that the amount of human correction required following application of a model will be approximately proportional with the overall size and diversity of the model's training dataset. *RIL-Contour* is designed to facilitate AID by (1) enabling deep-learning models to be applied to annotation images from within the software, (2) by providing mechanism from within the software to edit deep-learning derived annotations, (3) by providing a mechanism to export data to promote rapid model training, (4) by supporting concurrent workflows, and (5) by providing mechanisms which automate the sharing of deep-learning models between users of the software.

A limitation of *RIL-Contour* is the software has been designed to facilitate annotation of imaging stored in the Neuroimaging Informatics Technology Initiative (NIfTI) file format [39]. There are numerous tools (e.g., dcm2niix, MRIConvert) which can be used to convert DICOM imaging to the NIfTI file format. The NIfTI file format is a simpler format than the DICOM file format [39]. The NIfTI file format has been designed to encapsulate multi-dimensional imaging data within a single file. At present, there is a well-developed Python API to reliably read and write the file format, there are a number of medical imaging tools which read and write the format, and the format is extensively utilized within medical imaging research community [8–10, 12–14, 17, 24, 39]. A major limitation of the NIfTI file format is that it fails to capture much of the metadata commonly stored within DICOM files. To overcome this limitation, *RIL-Contour* supports the association of additional imaging metadata as a secondary CSV file and supports reading and writing this additional metadata from a MIRMAID content management system [24]. A focus of future development efforts is to add support in RIL-Contour to natively support datasets stored in DICOM.

*RIL-Contour* exports annotated voxel data as NIfTI files aligned to match the orientation and alignment of the source imaging. Additional non-imaging metadata is exported as tabular data in CSV and Excel format. These representations are programmatically convenient to work with. However, they do not facilitate broad data interoperability. The DICOM file format is capable of describing both imaging and metadata (contours, points, binary masks, and non-imaging data). The DICOM format is fully capable of encapsulating the metadata generated using RIL-Contour. A focus of future development efforts is to add support in *RIL-Contour* to export annotated datasets in the DICOM format to facilitate the utilization of *RIL-Contour* annotated datasets in other software packages.

## Conclusion

Deep-learning models are widely believed to require large training datasets for generalizable model convergence. The time required to annotate such datasets is a major barrier to the development of these models. We have developed the software *RIL-Contour* to accelerate medical imaging dataset annotation for deep learning. *RIL-Contour* provides annotation mechanisms designed to standardize annotation definitions and provides tools to easily apply deep-learning models to perform fully automated text and voxel annotation. *RIL-Contour* supports collaborative workflows and has been designed to accelerate annotation through the process of AID—a process through which deep-learning models are iteratively trained and utilized to generate draft annotation for a dataset that can then be edited as necessary.

## Software Availability

The source code for *RIL-Contour* and example deep-learning model plugins trained to identify the renal contrast enhancement phase of CT imaging and to perform patch-based kidney segmentation are publicly available on Gitlab at (https://gitlab.com/Philbrick/rilcontour). The software is distributed under a BSD style license. The software is provided “as is” and is intended for research purposes only. The software is installable using the Anaconda 3.6 package manager. License and installation instructions are available on Gitlab. The software is written in Python and utilizes common libraries for core functionality. Utilization of the machine-learning interface requires the additional installation of the OpenCV, Keras, and Tensorflow packages. The software is designed to work with data stored in the NIfTI format. Supplemental Python code has been published in the Gitlab archive demonstrating the use of dcm2nii to convert DICOM datasets to NIfTI. The *RIL-Contour* is broadly compatible with Python 2.7+ and Python 3.6+. Interaction with a MIRMAID content management system requires Python 2.7.

## References

[1] LeCun Y, Bengio Y, Hinton G (2015). Deep learning. Nature.

[2] Russakovsky O, Deng J, Su H, Krause J, Satheesh S, Ma S, Huang Z, Karpathy A, Khosla A, Bernstein M, Berg AC, Fei-Fei L (2015). ImageNet large scale visual recognition challenge. Int J Comput Vis.

[3] Weston AD, et al: Automated abdominal segmentation of CT scans for body composition analysis using deep learning*.* Radiology 181432, 2018 10.1148/radiol.2018181432 30526356

[4] Philbrick KA, Yoshida K, Inoue D, Akkus Z, Kline TL, Weston AD, Korfiatis P, Takahashi N, Erickson BJ (2018). What does deep learning see? Insights from a classifier trained to predict contrast enhancement phase from CT images. Am J Roentgenol.

[5] Korfiatis P, Kline TL, Lachance DH, Parney IF, Buckner JC, Erickson BJ (2017). Residual deep convolutional neural network predicts MGMT methylation status. J Digit Imaging.

[6] Akkus Z, Ali I, Sedlář J (2017). Predicting deletion of chromosomal arms 1p/19q in low-grade gliomas from MR images using machine intelligence. J Digit Imaging.

[7] Rajpurkar P, et al: Chexnet: Radiologist-level pneumonia detection on chest x-rays with deep learning*.* arXiv preprint arXiv:1711.05225, 2017

[8] Rueden CT (2017). ImageJ2: ImageJ for the next generation of scientific image data. BMC Bioinformatics.

[9] Kikinis R, Pieper SD, Vosburgh KG, Jolesz FA (2014). 3D Slicer: A platform for subject-specific image analysis, visualization, and clinical support. Intraoperative Imaging and Image-Guided Therapy.

[10] Kline TL, Edwards ME, Korfiatis P, Akkus Z, Torres VE, Erickson BJ (2016). Semiautomated segmentation of polycystic kidneys in T2-weighted MR images. Am J Roentgenol.

[11] Rubin DL, Willrett D, O’Connor MJ, Hage C, Kurtz C, Moreira DA (2014). Automated tracking of quantitative assessments of tumor burden in clinical trials. Transl Oncol.

[12] Yushkevich PA, Piven J, Hazlett HC, Smith RG, Ho S, Gee JC, Gerig G (2006). User-guided 3D active contour segmentation of anatomical structures: Significantly improved efficiency and reliability. NeuroImage.

[13] Fischl B (2012). FreeSurfer. NeuroImage.

[14] Papademetris X (2006). BioImage Suite: An integrated medical image analysis suite: An update. Insight J.

[15] Jiang H, van Zijl PCM, Kim J, Pearlson GD, Mori S (2006). DtiStudio: Resource program for diffusion tensor computation and fiber bundle tracking. Comput Methods Prog Biomed.

[16] McAuliffe MJ, et al: Medical image processing, analysis and visualization in clinical research. In: Proceedings 14th IEEE Symposium on Computer-Based Medical Systems. CBMS, 2001

[17] Jenkinson M, Beckmann CF, Behrens TEJ, Woolrich MW, Smith SM (2012). FSL. NeuroImage.

[18] Takahashi N, Sugimoto M, Psutka SP, Chen B, Moynagh MR, Carter RE (2017). Validation study of a new semi-automated software program for CT body composition analysis. Abdom Radiol.

[19] Carvalho LE, Sobieranski AC, von Wangenheim A: 3D segmentation algorithms for computerized tomographic imaging: A systematic literature review*.* J Digit Imaging 1–52, 2018 10.1007/s10278-018-0101-z PMC6261188 29915942

[20] Cheng J-Z, Ni D, Chou YH, Qin J, Tiu CM, Chang YC, Huang CS, Shen D, Chen CM (2016). Computer-aided diagnosis with deep learning architecture: Applications to breast lesions in US images and pulmonary nodules in CT scans. Sci Rep.

[21] Wachinger C, Reuter M, Klein T (2018). DeepNAT: Deep convolutional neural network for segmenting neuroanatomy. NeuroImage.

[22] Wang KC (2018). Standard lexicons, coding systems and ontologies for interoperability and semantic computation in imaging. J Digit Imaging.

[23] Agarwal V, Podchiyska T, Banda JM, Goel V, Leung TI, Minty EP, Sweeney TE, Gyang E, Shah NH (2016). Learning statistical models of phenotypes using noisy labeled training data. J Am Med Inform Assoc.

[24] Korfiatis PD, Kline TL, Blezek DJ, Langer SG, Ryan WJ, Erickson BJ (2015). MIRMAID: A content management system for medical image analysis research. RadioGraphics.

[25] Marcus DS, Olsen TR, Ramaratnam M, Buckner RL (2007). The extensible neuroimaging archive toolkit. Neuroinformatics.

[26] Kline TL, Korfiatis P, Edwards ME, Bae KT, Yu A, Chapman AB, Mrug M, Grantham JJ, Landsittel D, Bennett WM, King BF, Harris PC, Torres VE, Erickson BJ, CRISP Investigators (2017). Image texture features predict renal function decline in patients with autosomal dominant polycystic kidney disease. Kidney Int.

[27] Selvaraju RR, et al.: Grad-CAM: Why did you say that? arXiv [stat.ML], 2016

[28] Selvaraju RR, et al: Grad-cam: Visual explanations from deep networks via gradient-based localization. v3(8)7, 2016*.* See https://arxiv.org/abs/1610.02391

[29] Simonyan K, Vedaldi A, Zisserman A: Deep Inside Convolutional Networks: Visualising Image Classification Models and Saliency Maps. arXiv [cs.CV], 2013

[30] Zhou B, et al: Learning Deep Features for Discriminative Localization. arXiv [cs.CV], 2015

[31] Mehrtash A, et al: DeepInfer: Open-source deep learning deployment toolkit for image-guided therapy. In: Proceedings of SPIE--the International Society for Optical Engineering, 10135. 101351K, 2017 10.1117/12.2256011 PMC5467894 28615794

[32] Yu F, et al: Lsun: Construction of a large-scale image dataset using deep learning with humans in the loop*.* arXiv preprint arXiv:1506.03365, 2015

[33] Zhou Z, et al: Integrating active learning and transfer learning for carotid intima-media thickness video interpretation. 2018 10.1007/s10278-018-0143-2 PMC6456630 30402668

[34] Russakovsky O, Li L, Fei-Fei L: Best of both worlds: Human-machine collaboration for object annotation. In: 2015 IEEE Conference on Computer Vision and Pattern Recognition (CVPR). 2015

[35] Sakinis T, et al: Interactive segmentation of medical images through fully convolutional neural networks*.* arXiv preprint arXiv:1903.08205, 2019.

[36] Wu J, et al: Deep multiple instance learning for image classification and auto-annotation. In: 2015 IEEE Conference on Computer Vision and Pattern Recognition (CVPR). 2015

[37] Xu Y, et al: Deep learning of feature representation with multiple instance learning for medical image analysis. In: 2014 IEEE International Conference on Acoustics, Speech and Signal Processing (ICASSP). 2014

[38] Mettes P, Snoek CG, Chang S-F: Localizing actions from video labels and pseudo-annotations. arXiv preprint arXiv:1707.09143, 2017

[39] Li X, Morgan PS, Ashburner J, Smith J, Rorden C (2016). The first step for neuroimaging data analysis: DICOM to NIfTI conversion. J Neurosci Methods.

